# Nano and Giga Challenges in Electronics Photonics and Renewable Energy (NGC2011) Moscow-Zelenograd, Russia, September 12-16, 2011

**DOI:** 10.1186/1556-276X-7-326

**Published:** 2012-06-21

**Authors:** Anatoli Korkin

**Affiliations:** 1 Arizona State University, Phoenix, AZ, USA; 2 Oak Ridge National Laboratory, Oak Ridge, TN, USA; 3 Institute of Spectroscopy, Troitsk, Moskovskaya, Russia; 4 University of Milano-Bicocca, Milan, Italy

## 

This special issue of *Nanoscale Research Letters* is a collection of selected papers presented at the *Nano and Giga Challenges in Electronics, Photonics and Renewable Energy* (NGC2011) conference in Moscow and Zelenograd which addresses both theoretical and experimental achievements and provide a stimulating outlook for technological developments in these highly topical fields of research.

Informational (electronics and photonics) and renewable energy (solar systems, fuel cells and batteries) technologies have reached a new stage in their development – engineering limits in cost effective improvement of current technological approaches. The latest miniaturization of electronic devices is approaching atomic dimensions, interconnect bottlenecks are limiting circuit speeds, new materials are being introduced into microelectronics manufactured at an unprecedented rate, and alternative technologies to mainstream CMOS are being considered. The low cost of natural energy sources and ignorance of the limits and environmental impact from use of natural carbon based fuels have been long standing economic barriers to the development of alternative and more efficient solar systems, fuel cells and batteries. Nanotechnology is widely accepted as a source of potential solutions in securing future progress in information and energy technologies.

Academic and industrial researchers participated at NGC2011 conference presented tutorial, expository and original research papers dedicated to solving scientific and technological problems in electronics, photonics and renewable energy such as atomic scale materials design, bio- and molecular electronics, high frequency electronics, fabrication of nanodevices, magnetic materials and spintronics, materials and processes for integrated and subwave optoelectronics, nanoCMOS, new materials for FETs and other devices, nanoelectronics system architecture, nano optics and lasers, non-silicon materials and devices, quantum effects in devices, nano science and technology applications in development of novel solar energy devices, and fuel cells and batteries

Hosted by Moscow State University and NT-MDT, NGC2011 conference included the summer school (tutorial lectures), the symposium and a few satellite workshops. The first three meetings in the series of Nano & Giga Challenges conferences, NGCM2002 in Moscow, NGCM2004 in Krakow, and NGC2007 in Phoenix, were focused on the interdisciplinary research in electronics and photonics from the fundamentals of materials science to the development of new system architectures. Our last forum in Canada (NGC2009), which was held as a joint meeting with 14th Canadian Semiconductor Technology Conference (CSTC2009), expanded its topics to renewable energy research and development based on semiconductor and other materials including organic and biomolecular systems.

The success of the conference and publications would have not been possible without generous support from many sponsors and research institutions. The conference organizers gratefully acknowledge contributions and support from Russian Foundation for Basic Research, Russian Nanotechnology Corporation, Springer Publisher, INTEL, SEMATECH, STRF magazine and other sponsors and partners.

